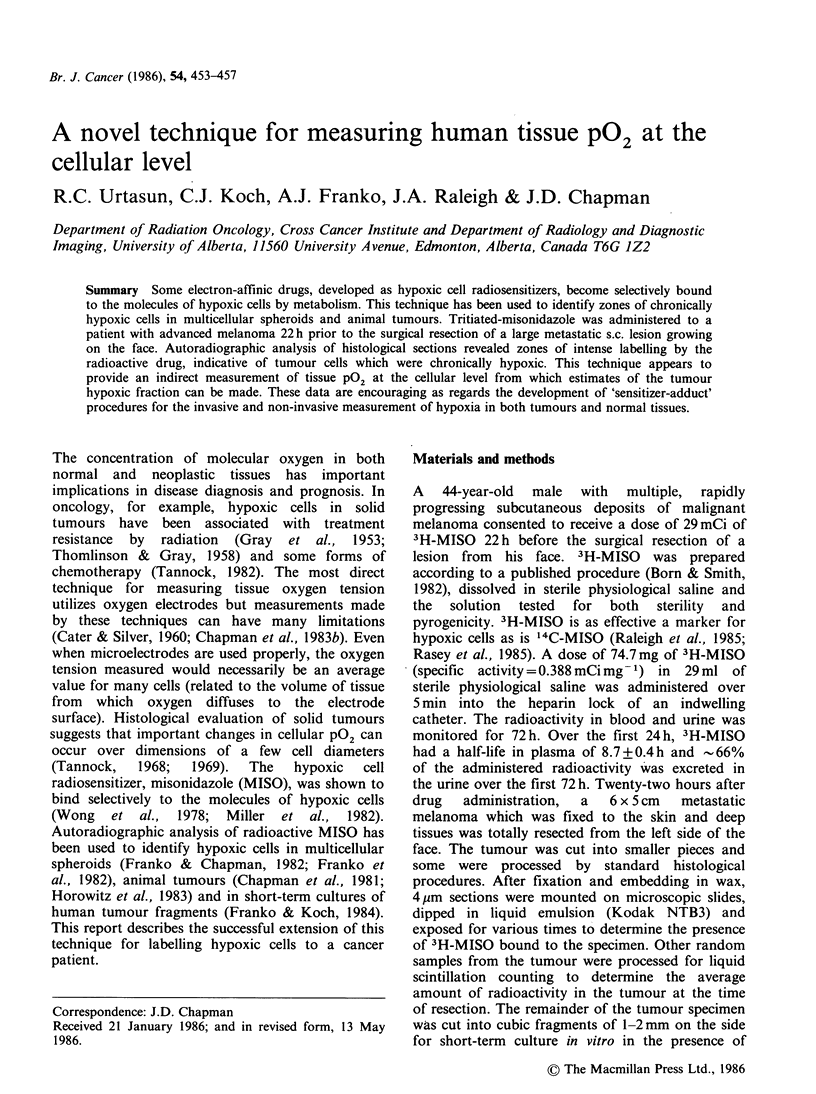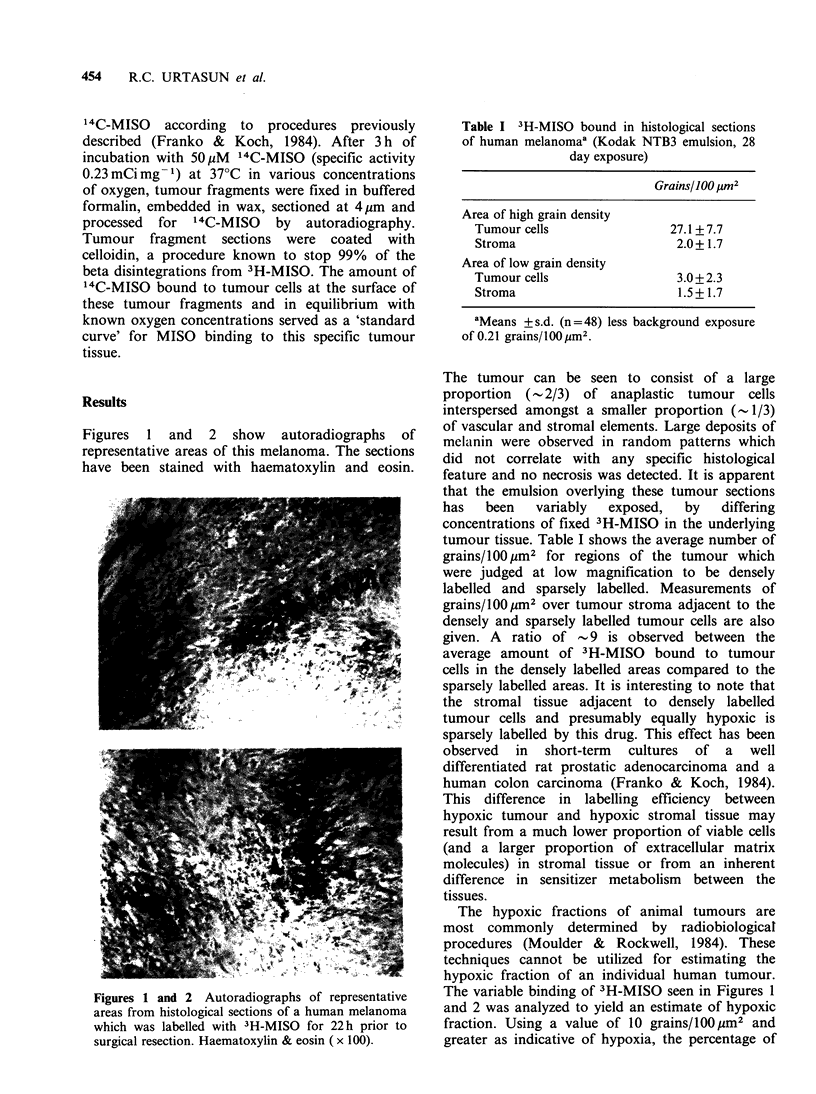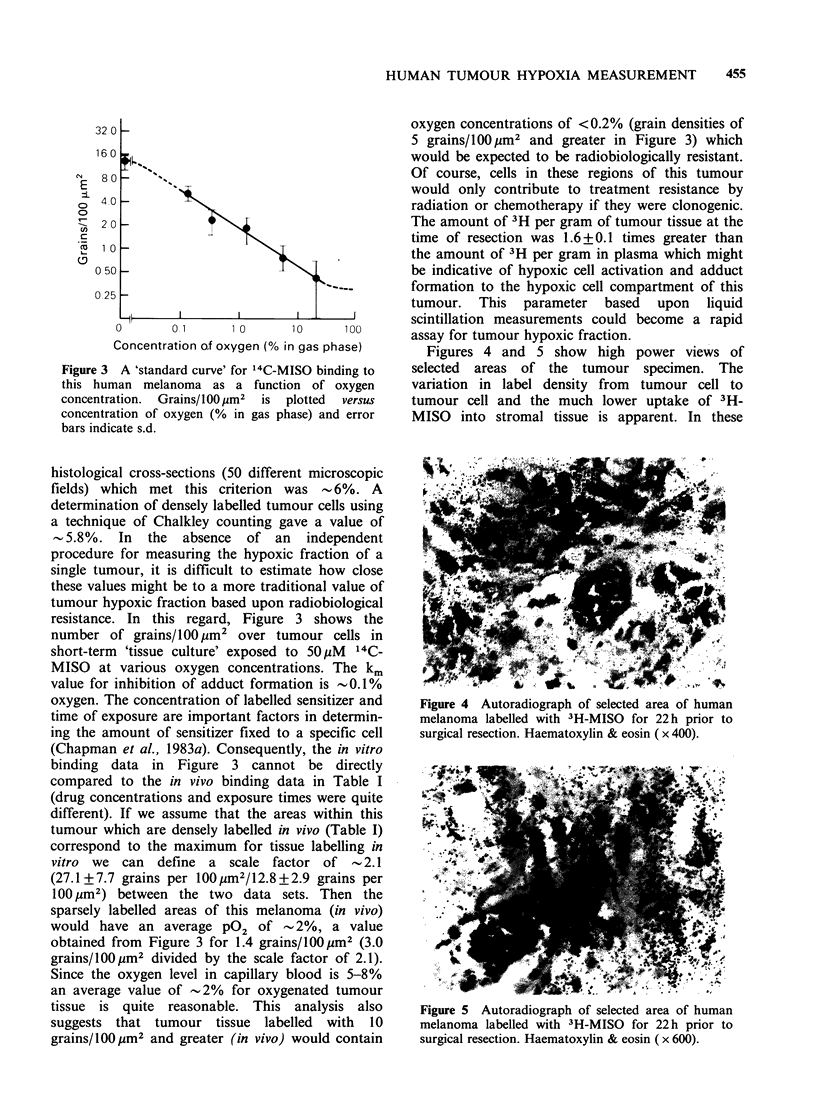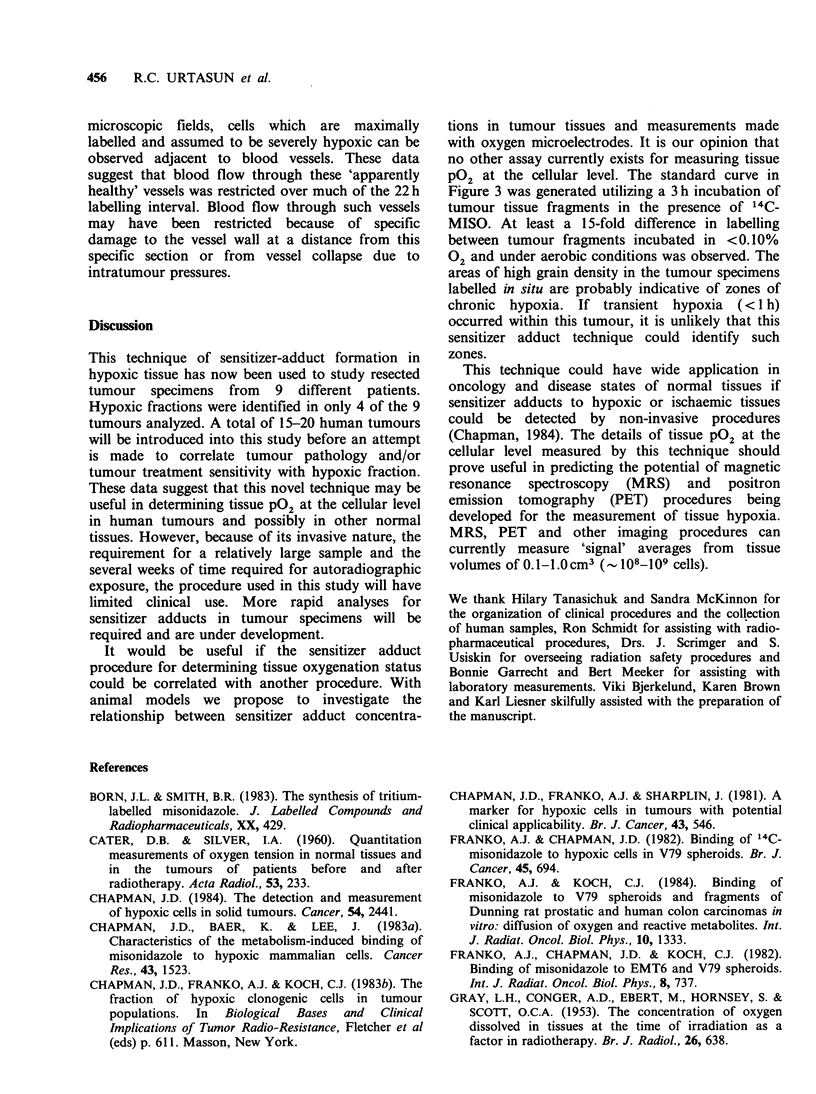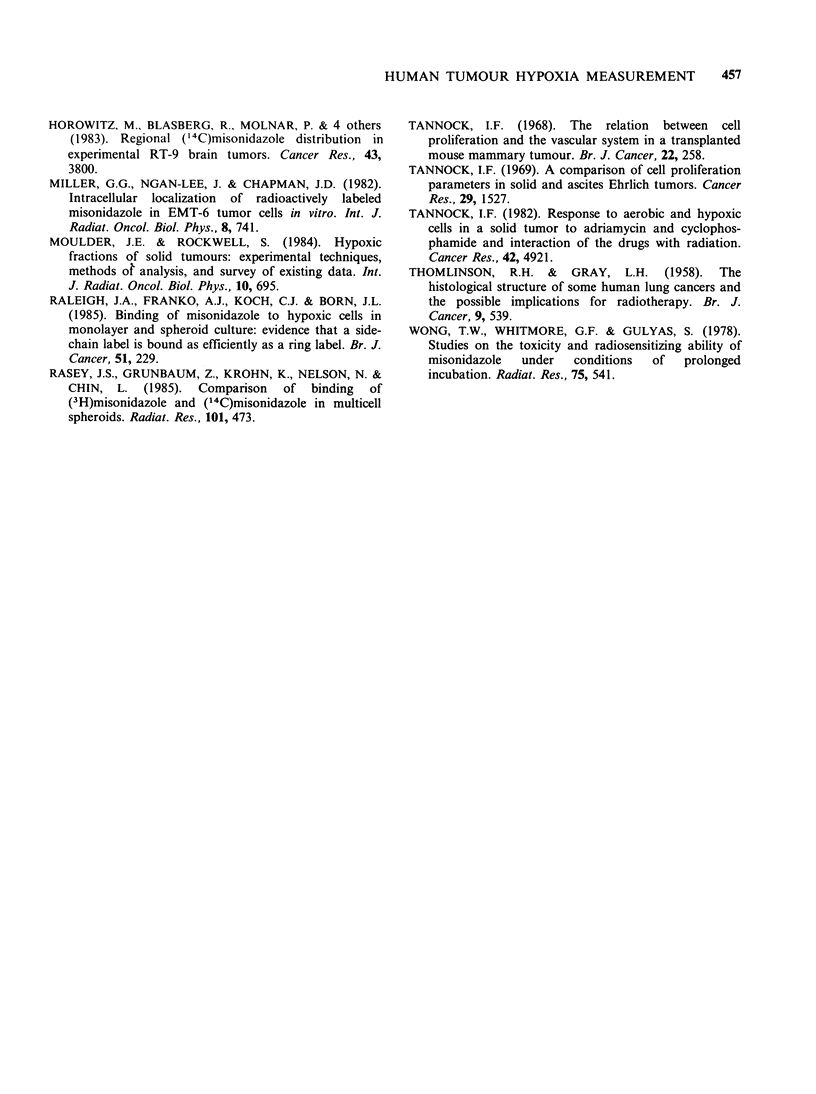# A novel technique for measuring human tissue pO2 at the cellular level.

**DOI:** 10.1038/bjc.1986.197

**Published:** 1986-09

**Authors:** R. C. Urtasun, C. J. Koch, A. J. Franko, J. A. Raleigh, J. D. Chapman

## Abstract

**Images:**


*Br. J. Cancer* (1986), **54**, 453–457

### A novel technique for measuring human tissue $pO_2$ at the cellular level

R.C. Urtasun, C.J. Koch, A.J. Franko, J.A. Raleigh & J.D. Chapman

*Department of Radiation Oncology, Cross Cancer Institute and Department of Radiology and Diagnostic Imaging, University of Alberta, 11560 University Avenue, Edmonton, Alberta, Canada T6G 1Z2*

**Summary** Some electron-affinic drugs, developed as hypoxic cell radiosensitizers, become selectively bound to the molecules of hypoxic cells by metabolism. This technique has been used to identify zones of chronically hypoxic cells in multicellular spheroids and animal tumours. Tritiated-misonidazole was administered to a patient with advanced melanoma 22 h prior to the surgical resection of a large metastatic s.c. lesion growing on the face. Autoradiographic analysis of histological sections revealed zones of intense labelling by the radioactive drug, indicative of tumour cells which were chronically hypoxic. This technique appears to provide an indirect measurement of tissue $pO_2$ at the cellular level from which estimates of the tumour hypoxic fraction can be made. These data are encouraging as regards the development of 'sensitizer-adduct' procedures for the invasive and non-invasive measurement of hypoxia in both tumours and normal tissues.

The concentration of molecular oxygen in both normal and neoplastic tissues has important implications in disease diagnosis and prognosis. In oncology, for example, hypoxic cells in solid tumours have been associated with treatment resistance by radiation (Gray *et al.*, 1953; Thomlinson & Gray, 1958) and some forms of chemotherapy (Tannock, 1982). The most direct technique for measuring tissue oxygen tension utilizes oxygen electrodes but measurements made by these techniques can have many limitations (Cater & Silver, 1960; Chapman *et al.*, 1983*b*). Even when microelectrodes are used properly, the oxygen tension measured would necessarily be an average value for many cells (related to the volume of tissue from which oxygen diffuses to the electrode surface). Histological evaluation of solid tumours suggests that important changes in cellular $pO_2$ can occur over dimensions of a few cell diameters (Tannock, 1968; 1969). The hypoxic cell radiosensitizer, misonidazole (MISO), was shown to bind selectively to the molecules of hypoxic cells (Wong *et al.*, 1978; Miller *et al.*, 1982). Autoradiographic analysis of radioactive MISO has been used to identify hypoxic cells in multicellular spheroids (Franko & Chapman, 1982; Franko *et al.*, 1982), animal tumours (Chapman *et al.*, 1981; Horowitz *et al.*, 1983) and in short-term cultures of human tumour fragments (Franko & Koch, 1984). This report describes the successful extension of this technique for labelling hypoxic cells to a cancer patient.

Correspondence: J.D. Chapman
Received 21 January 1986; and in revised form, 13 May 1986.

### Materials and methods

A 44-year-old male with multiple, rapidly progressing subcutaneous deposits of malignant melanoma consented to receive a dose of 29 mCi of $^3$H-MISO 22 h before the surgical resection of a lesion from his face. $^3$H-MISO was prepared according to a published procedure (Born & Smith, 1982), dissolved in sterile physiological saline and the solution tested for both sterility and pyrogenicity. $^3$H-MISO is as effective a marker for hypoxic cells as is $^{14}$C-MISO (Raleigh *et al.*, 1985; Rasey *et al.*, 1985). A dose of 74.7 mg of $^3$H-MISO (specific activity = 0.388 mCi mg$^{-1}$) in 29 ml of sterile physiological saline was administered over 5 min into the heparin lock of an indwelling catheter. The radioactivity in blood and urine was monitored for 72 h. Over the first 24 h, $^3$H-MISO had a half-life in plasma of $8.7 \pm 0.4$ h and $\sim 66\%$ of the administered radioactivity was excreted in the urine over the first 72 h. Twenty-two hours after drug administration, a $6 \times 5$ cm metastatic melanoma which was fixed to the skin and deep tissues was totally resected from the left side of the face. The tumour was cut into smaller pieces and some were processed by standard histological procedures. After fixation and embedding in wax, 4 $\mu$m sections were mounted on microscopic slides, dipped in liquid emulsion (Kodak NTB3) and exposed for various times to determine the presence of $^3$H-MISO bound to the specimen. Other random samples from the tumour were processed for liquid scintillation counting to determine the average amount of radioactivity in the tumour at the time of resection. The remainder of the tumour specimen was cut into cubic fragments of 1–2 mm on the side for short-term culture *in vitro* in the presence of

© The Macmillan Press Ltd., 1986

$^{14}C$-MISO according to procedures previously described (Franko & Koch, 1984). After 3h of incubation with 50 $\mu M$ $^{14}C$-MISO (specific activity 0.23 mCi mg$^{-1}$) at 37°C in various concentrations of oxygen, tumour fragments were fixed in buffered formalin, embedded in wax, sectioned at 4 $\mu m$ and processed for $^{14}C$-MISO by autoradiography. Tumour fragment sections were coated with celloidin, a procedure known to stop 99% of the beta disintegrations from $^{3}H$-MISO. The amount of $^{14}C$-MISO bound to tumour cells at the surface of these tumour fragments and in equilibrium with known oxygen concentrations served as a 'standard curve' for MISO binding to this specific tumour tissue.

### Results

Figures 1 and 2 show autoradiographs of representative areas of this melanoma. The sections have been stained with haematoxylin and eosin.

**Figures 1 and 2** Autoradiographs of representative areas from histological sections of a human melanoma which was labelled with $^{3}H$-MISO for 22h prior to surgical resection. Haematoxylin & eosin (×100).

**Table I** $^{3}H$-MISO bound in histological sections of human melanoma[a] (Kodak NTB3 emulsion, 28 day exposure)

| | *Grains/100 $\mu m^2$* |
|---|---|
| Area of high grain density | |
| Tumour cells | 27.1 ± 7.7 |
| Stroma | 2.0 ± 1.7 |
| Area of low grain density | |
| Tumour cells | 3.0 ± 2.3 |
| Stroma | 1.5 ± 1.7 |

[a]Means ± s.d. (n = 48) less background exposure of 0.21 grains/100 $\mu m^2$.

The tumour can be seen to consist of a large proportion (~2/3) of anaplastic tumour cells interspersed amongst a smaller proportion (~1/3) of vascular and stromal elements. Large deposits of melanin were observed in random patterns which did not correlate with any specific histological feature and no necrosis was detected. It is apparent that the emulsion overlying these tumour sections has been variably exposed, by differing concentrations of fixed $^{3}H$-MISO in the underlying tumour tissue. Table I shows the average number of grains/100 $\mu m^2$ for regions of the tumour which were judged at low magnification to be densely labelled and sparsely labelled. Measurements of grains/100 $\mu m^2$ over tumour stroma adjacent to the densely and sparsely labelled tumour cells are also given. A ratio of ~9 is observed between the average amount of $^{3}H$-MISO bound to tumour cells in the densely labelled areas compared to the sparsely labelled areas. It is interesting to note that the stromal tissue adjacent to densely labelled tumour cells and presumably equally hypoxic is sparsely labelled by this drug. This effect has been observed in short-term cultures of a well differentiated rat prostatic adenocarcinoma and a human colon carcinoma (Franko & Koch, 1984). This difference in labelling efficiency between hypoxic tumour and hypoxic stromal tissue may result from a much lower proportion of viable cells (and a larger proportion of extracellular matrix molecules) in stromal tissue or from an inherent difference in sensitizer metabolism between the tissues.

The hypoxic fractions of animal tumours are most commonly determined by radiobiological procedures (Moulder & Rockwell, 1984). These techniques cannot be utilized for estimating the hypoxic fraction of an individual human tumour. The variable binding of $^{3}H$-MISO seen in Figures 1 and 2 was analyzed to yield an estimate of hypoxic fraction. Using a value of 10 grains/100 $\mu m^2$ and greater as indicative of hypoxia, the percentage of

**Figure 3** A 'standard curve' for $^{14}$C-MISO binding to this human melanoma as a function of oxygen concentration. Grains/100 $\mu$m$^2$ is plotted *versus* concentration of oxygen (% in gas phase) and error bars indicate s.d.

histological cross-sections (50 different microscopic fields) which met this criterion was ~6%. A determination of densely labelled tumour cells using a technique of Chalkley counting gave a value of ~5.8%. In the absence of an independent procedure for measuring the hypoxic fraction of a single tumour, it is difficult to estimate how close these values might be to a more traditional value of tumour hypoxic fraction based upon radiobiological resistance. In this regard, Figure 3 shows the number of grains/100 $\mu$m$^2$ over tumour cells in short-term 'tissue culture' exposed to 50 $\mu$M $^{14}$C-MISO at various oxygen concentrations. The $k_m$ value for inhibition of adduct formation is ~0.1% oxygen. The concentration of labelled sensitizer and time of exposure are important factors in determining the amount of sensitizer fixed to a specific cell (Chapman *et al.*, 1983*a*). Consequently, the *in vitro* binding data in Figure 3 cannot be directly compared to the *in vivo* binding data in Table I (drug concentrations and exposure times were quite different). If we assume that the areas within this tumour which are densely labelled *in vivo* (Table I) correspond to the maximum for tissue labelling *in vitro* we can define a scale factor of ~2.1 ($27.1 \pm 7.7$ grains per 100 $\mu$m$^2$/$12.8 \pm 2.9$ grains per 100 $\mu$m$^2$) between the two data sets. Then the sparsely labelled areas of this melanoma (*in vivo*) would have an average $pO_2$ of ~2%, a value obtained from Figure 3 for 1.4 grains/100 $\mu$m$^2$ (3.0 grains/100 $\mu$m$^2$ divided by the scale factor of 2.1). Since the oxygen level in capillary blood is 5–8% an average value of ~2% for oxygenated tumour tissue is quite reasonable. This analysis also suggests that tumour tissue labelled with 10 grains/100 $\mu$m$^2$ and greater *(in vivo)* would contain oxygen concentrations of $<0.2\%$ (grain densities of 5 grains/100 $\mu$m$^2$ and greater in Figure 3) which would be expected to be radiobiologically resistant. Of course, cells in these regions of this tumour would only contribute to treatment resistance by radiation or chemotherapy if they were clonogenic. The amount of $^3$H per gram of tumour tissue at the time of resection was $1.6 \pm 0.1$ times greater than the amount of $^3$H per gram in plasma which might be indicative of hypoxic cell activation and adduct formation to the hypoxic cell compartment of this tumour. This parameter based upon liquid scintillation measurements could become a rapid assay for tumour hypoxic fraction.

Figures 4 and 5 show high power views of selected areas of the tumour specimen. The variation in label density from tumour cell to tumour cell and the much lower uptake of $^3$H-MISO into stromal tissue is apparent. In these

**Figure 4** Autoradiograph of selected area of human melanoma labelled with $^3$H-MISO for 22 h prior to surgical resection. Haematoxylin & eosin (×400).

**Figure 5** Autoradiograph of selected area of human melanoma labelled with $^3$H-MISO for 22 h prior to surgical resection. Haematoxylin & eosin (×600).

microscopic fields, cells which are maximally labelled and assumed to be severely hypoxic can be observed adjacent to blood vessels. These data suggest that blood flow through these 'apparently healthy' vessels was restricted over much of the 22 h labelling interval. Blood flow through such vessels may have been restricted because of specific damage to the vessel wall at a distance from this specific section or from vessel collapse due to intratumour pressures.

### Discussion

This technique of sensitizer-adduct formation in hypoxic tissue has now been used to study resected tumour specimens from 9 different patients. Hypoxic fractions were identified in only 4 of the 9 tumours analyzed. A total of 15–20 human tumours will be introduced into this study before an attempt is made to correlate tumour pathology and/or tumour treatment sensitivity with hypoxic fraction. These data suggest that this novel technique may be useful in determining tissue $pO_2$ at the cellular level in human tumours and possibly in other normal tissues. However, because of its invasive nature, the requirement for a relatively large sample and the several weeks of time required for autoradiographic exposure, the procedure used in this study will have limited clinical use. More rapid analyses for sensitizer adducts in tumour specimens will be required and are under development.

It would be useful if the sensitizer adduct procedure for determining tissue oxygenation status could be correlated with another procedure. With animal models we propose to investigate the relationship between sensitizer adduct concentrations in tumour tissues and measurements made with oxygen microelectrodes. It is our opinion that no other assay currently exists for measuring tissue $pO_2$ at the cellular level. The standard curve in Figure 3 was generated utilizing a 3 h incubation of tumour tissue fragments in the presence of $^{14}C$-MISO. At least a 15-fold difference in labelling between tumour fragments incubated in $<0.10\%$ $O_2$ and under aerobic conditions was observed. The areas of high grain density in the tumour specimens labelled *in situ* are probably indicative of zones of chronic hypoxia. If transient hypoxia ($<1$ h) occurred within this tumour, it is unlikely that this sensitizer adduct technique could identify such zones.

This technique could have wide application in oncology and disease states of normal tissues if sensitizer adducts to hypoxic or ischaemic tissues could be detected by non-invasive procedures (Chapman, 1984). The details of tissue $pO_2$ at the cellular level measured by this technique should prove useful in predicting the potential of magnetic resonance spectroscopy (MRS) and positron emission tomography (PET) procedures being developed for the measurement of tissue hypoxia. MRS, PET and other imaging procedures can currently measure 'signal' averages from tissue volumes of 0.1–1.0 $cm^3$ ($\sim 10^8$–$10^9$ cells).

We thank Hilary Tanasichuk and Sandra McKinnon for the organization of clinical procedures and the collection of human samples, Ron Schmidt for assisting with radio-pharmaceutical procedures, Drs. J. Scrimger and S. Usiskin for overseeing radiation safety procedures and Bonnie Garrecht and Bert Meeker for assisting with laboratory measurements. Viki Bjerkelund, Karen Brown and Karl Liesner skilfully assisted with the preparation of the manuscript.

### References

BORN, J.L. & SMITH, B.R. (1983). The synthesis of tritium-labelled misonidazole. *J. Labelled Compounds and Radiopharmaceuticals*, **XX**, 429.

CATER, D.B. & SILVER, I.A. (1960). Quantitation measurements of oxygen tension in normal tissues and in the tumours of patients before and after radiotherapy. *Acta Radiol.*, **53**, 233.

CHAPMAN, J.D. (1984). The detection and measurement of hypoxic cells in solid tumours. *Cancer*, **54**, 2441.

CHAPMAN, J.D., BAER, K. & LEE, J. (1983*a*). Characteristics of the metabolism-induced binding of misonidazole to hypoxic mammalian cells. *Cancer Res.*, **43**, 1523.

CHAPMAN, J.D., FRANKO, A.J. & KOCH, C.J. (1983*b*). The fraction of hypoxic clonogenic cells in tumour populations. In *Biological Bases and Clinical Implications of Tumor Radio-Resistance*, Fletcher et al (eds) p. 611. Masson, New York.

CHAPMAN, J.D., FRANKO, A.J. & SHARPLIN, J. (1981). A marker for hypoxic cells in tumours with potential clinical applicability. *Br. J. Cancer*, **43**, 546.

FRANKO, A.J. & CHAPMAN, J.D. (1982). Binding of $^{14}C$-misonidazole to hypoxic cells in V79 spheroids. *Br. J. Cancer*, **45**, 694.

FRANKO, A.J. & KOCH, C.J. (1984). Binding of misonidazole to V79 spheroids and fragments of Dunning rat prostatic and human colon carcinomas *in vitro:* diffusion of oxygen and reactive metabolites. *Int. J. Radiat. Oncol. Biol. Phys.*, **10**, 1333.

FRANKO, A.J., CHAPMAN, J.D. & KOCH, C.J. (1982). Binding of misonidazole to EMT6 and V79 spheroids. *Int. J. Radiat. Oncol. Biol. Phys.*, **8**, 737.

GRAY, L.H., CONGER, A.D., EBERT, M., HORNSEY, S. & SCOTT, O.C.A. (1953). The concentration of oxygen dissolved in tissues at the time of irradiation as a factor in radiotherapy. *Br. J. Radiol.*, **26**, 638.

HOROWITZ, M., BLASBERG, R., MOLNAR, P. & 4 others (1983). Regional ($^{14}$C)misonidazole distribution in experimental RT-9 brain tumors. *Cancer Res.*, **43,** 3800.

MILLER, G.G., NGAN-LEE, J. & CHAPMAN, J.D. (1982). Intracellular localization of radioactively labeled misonidazole in EMT-6 tumor cells *in vitro*. *Int. J. Radiat. Oncol. Biol. Phys.*, **8,** 741.

MOULDER, J.E. & ROCKWELL, S. (1984). Hypoxic fractions of solid tumours: experimental techniques, methods of analysis, and survey of existing data. *Int. J. Radiat. Oncol. Biol. Phys.*, **10,** 695.

RALEIGH, J.A., FRANKO, A.J., KOCH, C.J. & BORN, J.L. (1985). Binding of misonidazole to hypoxic cells in monolayer and spheroid culture: evidence that a side-chain label is bound as efficiently as a ring label. *Br. J. Cancer*, **51,** 229.

RASEY, J.S., GRUNBAUM, Z., KROHN, K., NELSON, N. & CHIN, L. (1985). Comparison of binding of ($^{3}$H)misonidazole and ($^{14}$C)misonidazole in multicell spheroids. *Radiat. Res.*, **101,** 473.

TANNOCK, I.F. (1968). The relation between cell proliferation and the vascular system in a transplanted mouse mammary tumour. *Br. J. Cancer*, **22,** 258.

TANNOCK, I.F. (1969). A comparison of cell proliferation parameters in solid and ascites Ehrlich tumors. *Cancer Res.*, **29,** 1527.

TANNOCK, I.F. (1982). Response to aerobic and hypoxic cells in a solid tumor to adriamycin and cyclophosphamide and interaction of the drugs with radiation. *Cancer Res.*, **42,** 4921.

THOMLINSON, R.H. & GRAY, L.H. (1958). The histological structure of some human lung cancers and the possible implications for radiotherapy. *Br. J. Cancer*, **9,** 539.

WONG, T.W., WHITMORE, G.F. & GULYAS, S. (1978). Studies on the toxicity and radiosensitizing ability of misonidazole under conditions of prolonged incubation. *Radiat. Res.*, **75,** 541.